# Two Plant Bacteria, *S. meliloti* and *Ca.* Liberibacter asiaticus, Share Functional *znuABC* Homologues That Encode for a High Affinity Zinc Uptake System

**DOI:** 10.1371/journal.pone.0037340

**Published:** 2012-05-24

**Authors:** Cheryl M. Vahling-Armstrong, Huasong Zhou, Lesley Benyon, J. Kent Morgan, Yongping Duan

**Affiliations:** United States Horticultural Research Laboratory, Plant Pathology, United States Department of Agriculture Agricultural Research Service, Fort Pierce, Florida, United States of America; University of Wisconsin-Milwaukee, United States of America

## Abstract

The Znu system, encoded for by *znuABC*, can be found in multiple genera of bacteria and has been shown to be responsible for the import of zinc under low zinc conditions. Although this high-affinity uptake system is known to be important for both growth and/or pathogenesis in bacteria, it has not been functionally characterized in a plant-associated bacterium. A single homologue of this system has been identified in the plant endosymbiont, *Sinorhizobium meliloti*, while two homologous systems were found in the destructive citrus pathogen, *Candidatus* Liberibacter asiaticus. To understand the role of these protein homologues, a complementation assay was devised allowing the individual genes that comprise the system to be assayed independently for their ability to reinstate a partially-inactivated Znu system. Results from the assays have demonstrated that although all of the genes from *S. meliloti* were able to restore activity, only one of the two *Ca.* Liberibacter asiaticus encoded gene clusters contained genes that were able to functionally complement the system. Additional analysis of the gene clusters reveals that distinct modes of regulation may also exist between the *Ca.* Liberibacter asiaticus and *S. meliloti* import systems despite the intracellular-plant niche common to both of these bacteria.

## Introduction

The ability to import zinc is critical for many species of bacteria because of its use as an enzymatic cofactor or structural element in many cellular proteins [1]. Since zinc is both highly charged and hydrophilic, it cannot cross the bacterial membrane via passive diffusion [2] and must gain entrance into the bacterial cells though the actions of either non-specific or specific transport systems [3]. An example of each of the two types of systems for zinc import is that encoded by *pit* and *znu,* respectively [4]. The non-specific *pit* system is constitutively expressed and has a low affinity for zinc in contrast to the specific transport system encoded by the *znu* genes.

The Znu (zinc uptake) system has a high affinity for zinc and was discovered in *Escherichia coli* during a transposon screen with *lacZ* gene cluster fusions [5]. This system has since been classified as a member of the ATP-binding cassette (ABC) transporter family and is composed of three protein products: ZnuA, ZnuB, and ZnuC. ZnuA functions as a metallochaperone and is responsible for binding zinc within the periplasm. ZnuB acts as an integral membrane permease and works in concert with ZnuC. ZnuC is the ATPase subunit of the ABC transporter, which provides energy for the transport process through ATP hydrolysis. In *E. coli*, the zinc uptake regulator, or *zur*, appears to bind to and repress the promoters of both *znuA* and *znuCB,* thus providing a mechanism for zinc regulation within the cell [5]. Although the Znu system has been shown to be important for both growth and/or pathogenesis in several bacterium such as *Synechocystis* [6], *Pasteurella multocida* [7], and *Salmonella enterica* [8], it has not been characterized in plant-associated bacteria.

*Sinorhizobium meliloti* and *Candidatus* Liberibacter asiaticus are two closely related members of the Rhizobiaceae family with drastically different genome sizes [9] that can be found living in both similar and distinct ecological niches. *S. meliloti* is both a free-living soil microorganism and a plant-associated bacterium, which is known for producing nitrogen-fixing nodules on leguminous plants (for a review see [10]). The symbiosis takes place under nitrogen limiting conditions when the bacteria are internalized by the plant’s roots and are subsequently able to convert atmospheric nitrogen into ammonia, which then acts as a source of nitrogen for the plant. In 2001, the completed sequence of both the chromosome and the two megaplasmids of *S. meliloti* became publicly available [11], [12], [13]. Since that time, tools allowing genome-wide screening have been developed [14], [15], [16] in an effort to understand the symbiotic relationship between the plant and the bacterium on a molecular level.

*Ca.* Liberibacter asiaticus is one of three different species of Liberibacter that cause the devastating disease of citrus plants known as huanglongbing (HLB) or citrus greening (for a review see [17], [18]). This fastidious bacterium resides within the sieve tube elements [19] and is transmitted by Asian citrus psyllids (*Diaphorina citri*) that have fed on the phloem of infected plants. Symptoms characteristic of HLB disease include blotchy mottle, vein corking, and leaf chlorosis in addition to other symptoms that resemble those of a zinc nutritional deficiency. Although no adequate control measures are currently available for this disease, the recent publication of the *Ca.* Liberibacter asiaticus genome sequence may provide insight into areas amenable to the development of novel control strategies for HLB disease [9].

In this report we have characterized the *znu* gene cluster(s) and their involvement in the high-affinity uptake of zinc in two closely related plant-associated intracellular bacteria, *S. meliloti* and *Ca.* Liberibacter asiaticus. The putative genes comprising the Znu system of *S. meliloti* along with that of *Ca.* Liberibacter asiaticus were tested individually for their ability to restore the high-affinity zinc uptake system in knock-out strains of both *E. coli* and *S. meliloti*. In addition, a bioinformatic analysis of the gene clusters was used to uncover putative modes of regulation for the import systems.

## Materials and Methods

### Bacterial Strains and Media

*S. meliloti* strain Sm1021, which was obtained from Brenda K. Schroeder at Washington State University, was grown at 28°C in either Luria-Bertani (LB) broth, or in tryptone-yeast (TY) media. The wild-type *Escherichia coli* strain BW25113, along with its Δ*znu* derivatives JW1847-1, JW1848-1, and JW5831-1 [20] were obtained from the *E. coli* Genetic Stock Center at Yale University **(** **Table 1**). *E. coli* Top10 cells (Invitrogen, Carlsbad, CA) were used as a host for plasmid construction. *E. coli* strains were grown at 37°C in either LB broth, on LB agar plates, or in TY media supplemented with the following concentrations of antibiotics: 50 µg/mL of ampicillin, and 50 µg/mL of kanamycin.

**Table 1:** Plasmids and strains used in this study.

Name	Relevant Characteristic(s)	Reference
BW25113	Wild-type *E. coli* strain used in the construction of the Δ*znu* strains	[20]
DH5α	Strain used for cloning of constructs	Invitrogen (Carlsbad, CA)
JW1847-1	Δ*znuC* derivative of BW25113 strain	[20]
JW1848-1	Δ*znuB* derivative of BW25113 strain	[20]
JW5831-1	Δ*znuA* derivative of BW25113 strain	[20]
Sm1021	Wild-type *S. meliloti* 1021 strain	(Meade, 1982)
pASK-IBA3	Expression plasmid containing the tetracycline promoter	IBA (Gottingen, Germany)
pBBR1MC-5	Broad host range cloning cosmid	[25]
pBB:La	*znuA* gene from *Ca.* Liberibacter asiaticus gene cluster 1 in the broad host range cloning cosmid	This study
pBB:Lb	*znuB* gene from *Ca.* Liberibacter asiaticus gene cluster in the broad host range cloning cosmid	This study
pBB:Lc	*znuC* gene from *Ca.* Liberibacter asiaticus gene cluster 1 in the broad host range cloning cosmid	This study
pBB:Ra	*znuA* gene from *S. meliloti* 1021 in the broad host range cloning cosmid	This study
pBB:Rb	*znuB* gene from *S. meliloti* 1021 in the broad host range cloning cosmid	This study
pBB:Rc	*znuC* gene from *S. meliloti* 1021 in the broad host range cloning cosmid	This study
pCMV219	*znuA* gene from *S. meliloti* 1021 under the inducible tetracycline promoter	This study
pCMV220	*znuB* gene from *S. meliloti* 1021 under the inducible tetracycline promoter	This study
pCMV221	*znuC* gene from *S. meliloti* 1021 under the inducible tetracycline promoter	This study
pCR2.1	TOPO TA cloning vector	Invitrogen (Carlsbad, CA)
pESMc04243	Plasmid containing the *znuB* gene from *S. meliloti* 1021	[16]
pESMc04244	Plasmid containing the *znuC* gene from *S. meliloti* 1021	[16]
pESMc04245	Plasmid containing the *znuA* gene from *S. meliloti* 1021	[16]
pK19mobGII	Mobilizable vector used for gene replacement in *S. meliloti* 1021	[23]
pRK2073	Triparental mating helper plasmid	[24]
pyebI	*znuB* gene from *E.coli* under the inducible tetracycline promoter	This study
pyebL	*znuA* gene from *E.coli* under the inducible tetracycline promoter	This study
pyebM	*znuC* gene from *E.coli* under the inducible tetracycline promoter	This study
pznuA(05)	*znuA* gene from *Ca.* Liberibacter asiaticus gene cluster 1 under the inducible tetracycline promoter	This study
pznuA(12)	*znuA* gene from *Ca.* Liberibacter asiaticus gene cluster 2 under the inducible tetracycline promoter	This study
pznuB(05)	*znuB* gene from *Ca.* Liberibacter asiaticus gene cluster 1 under the inducible tetracycline promoter	This study
pznuB(12)	*znuB* gene #1 from *Ca.* Liberibacter asiaticus gene cluster 2 under the inducible tetracycline promoter	This study
pznuB_2(12)	*znuB* gene #2 from *Ca.* Liberibacter asiaticus gene cluster 2 under the inducible tetracycline promoter	This study
pznuC(05)	*znuC* gene from *Ca.* Liberibacter asiaticus gene cluster 1 under the inducible tetracycline promoter	This study
pznuC(12)	*znuC* gene from *Ca.* Liberibacter asiaticus gene cluster 2 under the inducible tetracycline promoter	This study

### Sequence Analysis

The percent sequence identity was determined using the ClustalW method in the program AlignX (Vector NTI Advance 11.0). Signal sequences were predicted by both SignalP 3.0 (http://www.cbs.dtu.dk/services/SignalP/) and SIG-Pred (http://bmbpcu36.leeds.ac.uk/prot_analysis/Signal.html). The ProtParam tool on the ExPASy server was used to predict both the molecular weight and isoelectric point of the proteins [21]. The HMMTOP server (http://www.enzim.hu/hmmtop/index.html) was used to predict the transmembrane topology of the proteins.

### Reverse Transcription PCR

**Figure 1:**
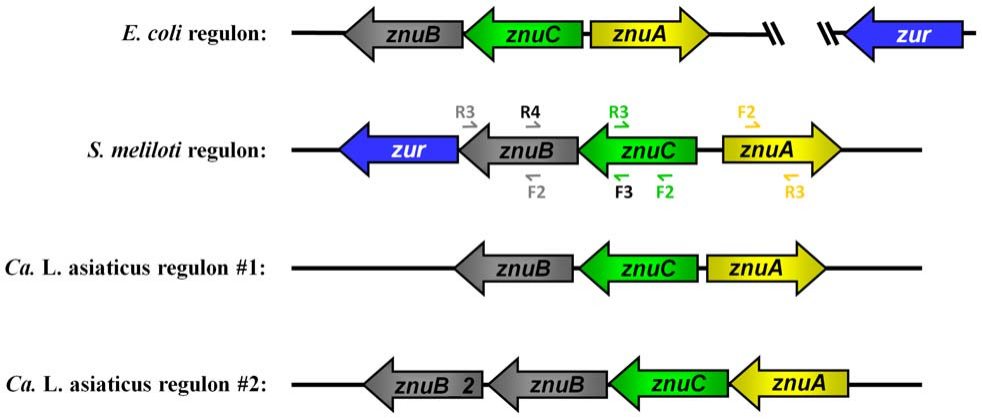
Schematic comparison of the Znu gene cluster from *E. coli, S. meliloti* and *Ca.* Liberibacter asiaticus. Znu homologues have been identified in the two intracellular-plant bacterium, *S. meliloti* and *Ca.* Liberibacter asiaticus. The arrangement of *znuA, znuB,* and *znuC* genes in each bacterium are shown. A BLAST search of the completed genomes revealed that only the *E. coli* and *S. meliloti* gene clusters contain the zinc uptake regulator, *zur.* In *E. coli,* the *zur* gene is located 2.2 Mb downstream of *znuA* while in *S. meliloti* it is immediately upstream of *znuB*. Primers are depicted as small arrows and are colored coded to represent the genes in which they are located. Notations above the primers correspond to the final letters in the primer name and are colored according to the genes that they amplify. Names of primers that were used to cross the junction of a gene are listed in black.

The operon structure of the Znu system in *S. meliloti* 1021 was determined using reverse transcription PCR. Total RNA was extracted from the bacterial cultures grown at 25°C for 72 hours (hrs) in LB broth (Sigma, St. Louis, MO) using TRI Reagent (Sigma) according to the manufacture’s protocol. Subsequent to RNA isolation, each sample was treated with RQ1 DNase (Promega, Madison, WI) to remove any contaminating DNA and verified DNA free by PCR. Two step reverse transcription PCR (RT-PCR) analyses were performed with gene specific primers, which were designed to span the junction between the genes **(Table S1 and** **Fig. 1** **)**, using the GoScript Reverse Transcription System (Promega) per manufacturer’s instructions. The PCR profile consisted of a 3 min. denaturation at 95°C, followed by 40 cycles of 95°C for 30 sec., 57°C for 30 sec., and 72°C for 1 min. The final PCR step was an incubation period of 5 min at 72°C before being held at 4°C. Genomic DNA extracted from *S. meliloti* 1021 was used as a positive control for the PCR reactions. Amplicon products were electrophoresed on 2% agarose gels and visualized with ethidium bromide staining. A Logic 200 Imaging System from Kodak with Kodak Molecular Imaging Software v. 4.0.5 (Eastman Kodak Company, Rochester, NY) was used for gel image capture.

### DNA Manipulations

PCR amplification of the *znu* genes for cloning was performed with Platinum Taq High Fidelity Polymerase (Invitrogen), while all subsequent PCR amplifications were performed with Taq DNA Polymerase (New England Biolabs, Ipswich, MA). Resulting DNA products were purified from agarose gels using the QIAquick gel extraction kit (Qiagen, Valencia, CA), while restriction fragments were purified with the QIAquick PCR purification system (Qiagen). Genomic DNA was extracted from *E. coli* strain BW25113 by boiling a sample from an overnight culture, pelleting the cell debris, and using the soluble extract for PCR. Plasmid DNA was isolated from *E. coli* cultures using the QIAprep Spin Miniprep kit (Qiagen). DNA sequencing was done by the U.S. Horticulture Research Laboratory Core Facility using Big Dye version 3.1 and the 3730×l DNA analyzer (Applied Biosystems, Carlsbad, CA).

### Construction of Plasmids Containing the *znuA*, *znuB* or *znuC* Genes from *Ca.* Liberibacter Asiaticus, *E. coli*, and *S. meliloti*

Individual genes from the two *znuABC* gene clusters in *Ca.* Liberibacter asiaticus, *S. meliloti*, and *E. coli* were placed on a plasmid under the control of the tetracycline inducible promoter as follows: each *znu* gene was amplified from *Ca.* Liberibacter asiaticus genomic DNA isolated from *Ca.* Liberibacter asiaticus-infected psyllids [22] using primers specific for the gene of interest **(Table S1**), while the genes from *E. coli* were amplified from BW25113 genomic DNA and those from *S. meliloti* were amplified with gene specific primers from plasmids pESMc04245, pESMc04243, and pESMc04244, respectively [16]. The amplified products were purified on 1% agarose gels and both the PCR products and the vector, pASK-IBA3 (IBA Biotagnology, Goettingen, Germany), were subsequently digested with BsaI (New England Biolabs) using the engineered restriction sites. T4 DNA ligase (New England Biolabs) was used to ligate the resulting fragments to the vector producing pznuA(05), pznuA(12), pznuB(05), pznuB(12), pznuB_2(12), pznuC(05), pznuC(12), pyebL, pyebI, pyebM, pCMV219, pCMV220, and pCMV221 **(** **Table 1**). The clones were verified via DNA sequencing and transformed into the corresponding *E. coli znu* knock-out strains.

### Complementation Assay of *Δznu E. coli* Strains with Tetracycline-inducible Gene Expression Plasmids

Growth curves for the complementation assay of the *Δznu E. coli* strains were produced by using 5 µL of a culture grown overnight in TY to inoculate 10 mL of fresh TY or TY containing 0.4 mM EDTA. Media for complementation assays also contained 50 µg/mL ampicillin, 50 µg/mL kanamycin, and 50 µg/L anhydrotetracycline (AHT) for the induction of the *znu* genes on the complementing plasmids. Growth was measured at the time points indicated using a Biomate 3 spectrophotometer (Thermo Electron Corp.) at an absorbance of 600 nm (OD_600_).

### Growth of Wild-type *E. coli* and *S. meliloti* in EDTA with and without Zinc Repletion

Growth inhibition studies were conducted on wild-type *E. coli and S. meliloti* cultures by growing them in TY media containing an increasing concentration of EDTA (pH 8.0), ranging from 0 mM to 3.2 mM. These cultures were inoculated with a 1∶100 dilution of an overnight growth of the wild-type strains. After approximately 20 hrs of growth, the OD_600_ of these strains were measured and recorded.

For zinc repletion experiments, wild-type *S. meliloti* 1021 cultures were grown in TY media containing 0, 0.1, 0.2, or 0.4 mM of EDTA (pH 8.0). Different concentrations of ZnSO_4_, ranging from 0 mM to 0.5 mM, was added to the culture media in order to replete the zinc that was chelated by the EDTA. Cultures were inoculated and the growth recorded as above.

### Generation of *S. meliloti znu* Deletion Mutants

Partial sequences of the three *znu* genes of *S. meliloti* were amplified from genomic DNA using the forwards (For) and reverse (Rev) primer pairs ZnuA.M, ZunB.M and ZunC.M, respectively. The resulting PCR products were ligated into pCR2.1 (Invitrogen), digested with *EcoR*I, and ligated into the mobilizable vector pK19mobGII [23]. The constructs were transformed into DH5α, and subsequently introduced into wild type *S. meliloti* 1021 by triparental mating with the helper pRK2073 for mutant isolation [24]. For complementation analysis, *znu* genes from *S. meliloti* and *Ca.* Liberibacter asiaticus were cloned into cosmid pBBR1MC-5 [25], and introduced into the corresponding mutant by triparental mating. The ability of each strain to transport zinc was tested as described above except the OD_600_ of the cultures were measured and recorded after 48 hrs of growth at 28°C.

## Results

### Structure of the Znu Gene Cluster in *S. meliloti* and *Ca. *Liberibacter Asiaticus

**Figure 2:**
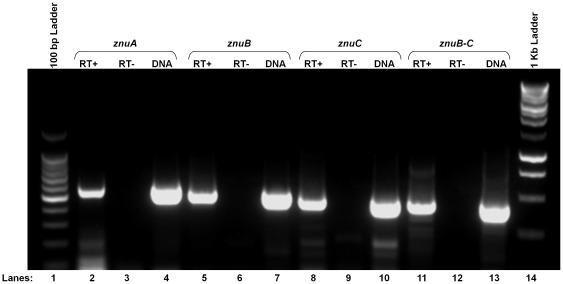
Operon structure of the *znu* genes in *S. meliloti*1021. Reverse transcription PCR was performed on total RNA isolated from *S. meliloti* to determine if the *znuB* and *znuC* genes were co-transcribed. Primer sets were designed to amplify fragments either internal to *znuA* (lanes 2–4), *znuB* (lanes 5–7),and *znuC* (lanes 8–10) or a fragment that span the junction between *znuB-C* (lanes 11–13). Reverse transcriptase was added to the reaction in lanes 2, 5, 8, and 11 (RT+) but omitted from lanes 3, 6, 9, and 12 (RT-) to demonstrate that the RNA was free of any detectable DNA contaminations. Genomic DNA was also amplified with the corresponding primers for use as a positive control (lanes 4, 7, 10, and 13). Experiment shown is representative of data from three independent replicates.

The annotation of the *S. meliloti* genome sequence reveals a putative gene cluster with some similarities to the high-affinity zinc uptake system (*znuABC)* in *E. coli*. The arrangement of the genes in the *S. meliloti* cluster resembles that of *E. coli* with the exception of the zinc uptake regulator, *zur,* which is located immediately upstream of *znuB* in *S. meliloti* but is approximately 2.2 Mb downstream of *znuA* in *E. coli* **(** **Fig. 1** **)**. To determine if the *znuCB* genes are co-transcribed in *S. meliloti* as they are in other bacteria [7], reverse transcription PCR was performed using RNA extracted from an axenic culture. A primer set was designed to span the junction between the genes of the putative operon **(** **Fig. 1** **and Table S1)** so that a product would be seen only if a single transcript encoded the adjacent open reading frames (ORFs). In this assay, a 456 bp product was detected **(** **Fig. 2** **)**, which corresponds to the distance between the primers on either side of the junction and indicates that *znuC* and *znuB* are indeed co-transcribed. Primer sets designed to amplify a region within each gene (583 bp for *znuA*, 526 bp for *znuB,* and 473 bp for *znuC*) were also used. Fragments corresponding to the individual genes were also detected, demonstrating the presence of transcript for all three genes under the conditions used.

Within the *Ca.* Liberibacter asiaticus genome, two putative gene clusters with similarity to *znuABC* of *E. coli* were identified. These gene clusters are located approximately 191 Kb apart on the chromosome and contain either three or four genes consisting of *znuACB* or *znuACBB,* respectively **(** **Fig. 1** **)**. The gene arrangement of the first *Ca.* Liberibacter asiaticus gene cluster suggests that the *znuA* gene and the *znuCB* genes are divergently transcribed, whereas in the second *Ca.* Liberibacter asiaticus gene cluster, all of the *znu* genes appear to be co-transcribed. Because of the difficulty with its cultivation and the inability to obtain a substantial quantity of viable cells in culture [26], efforts were made to determine the operon structure of the *Ca.* Liberibacter asiaticus genes by extracting bacterial RNA from infected plant tissue. Despite several attempts, conclusive results could not be obtained (data not shown). Additional bioinformatic analysis using the basic local alignment search tool (BLAST) to query the completed genome of *Ca.* Liberibacter *asiaticus* did however reveal the lack of a gene within the *Ca.* Liberibacter asiaticus sequence that is homologous to the negative regulator, *zur,* from *E. coli*.

### Sequence Analysis of Putative Znu Systems Supports Proper Structure and Localization of Protein Components

Sequence analysis of the individual components composing the Znu system supports the hypothesis that the *Ca.* Liberibacter asiaticus and *S. meliloti* proteins are functional even though the percent identity amongst species remains low **(** **Table 2** **)**. For example, the size of the coding sequence for the *znuA*, *znuB* and *znuC* genes and the molecular weight of the corresponding protein products from both bacteria are comparable to that of the homologous genes/proteins in *E. coli*. Although the isoelectric point (pI) of most of the predicted proteins are similar to their *E. coli* homologues, the pI of one of the ZnuC proteins from *Ca. *Liberibacter asiaticus (pI of 5.8) and the ZnuC protein from *S. meliloti* (pI of 6.4), are vastly different from the pI of 9.4 predicted for the *E. coli* ZnuC. Nevertheless, all of the predicted ZnuC proteins contain both the Walker A motif (GX_2_GXGKT/S) and Walker B motif (R/KX_(7−8)_hhhhD) where X represents any amino acid and h represents any hydrophobic residue. These motifs are involved in ATP binding and are common to ATPases, which is indicative of their function in the ABC transporter. In addition, the HMMTOP server [27] predicts seven transmembrane helices for all of the ZnuB proteins except that of ZnuB from the second gene cluster in *Ca.* Liberibacter asiaticus, in which only six are predicted. The presence of these domains is in accordance with their predicted role as the membrane permease of the ABC transporter. Signal peptide prediction tools (SIG-Pred and SignalP 3.0) showed that ZnuA from both *Ca.* Liberibacter asiaticus and *S. meliloti* contain signal sequences, which suggests that the proteins are targeted to the periplasmic space and provides an additional line of evidence supporting the prediction that ZnuA acts as a metallochaperone in the system.

**Table 2:** Bioinformatic analysis of the components comprising the Znu gene clusters.

Organism	Gene	GI Number^a^	Length (bp)	Molecular Weight (KDa)	Identity^b^ (%)	pI
***E. coli*** ** K-12**	*znuA*	87081990	933	33.8	100	5.6
	*znuB*	1788166	786	27.7	100	8.7
	*znuC*	1788165	756	27.9	100	9.4
***Ca.*** ** Liberibacter asiaticus**	*znuA* #1	254040394	885	33.4	29	6.7
	*znuB* #1	254040396	783	28.7	39	8.6
	*znuC* #1	254040395	723	26.7	42	9.4
	*znuA* #2	254040214	885	33.2	17	6.3
	*znuB* #2	254040216	822	30.1	18	8.9
	*znuB_2* #2	254040217	834	30.3	17	8.8
	*znuC* #2	254040215	843	31.3	23	5.8
***S. meliloti 1021***	*znuA*	15074849	1023	35.9	34	4.9
	*znuB*	15074847	828	29.2	47	6.7
	*znuC*	15074848	876	31.6	41	6.4

a In accordance with the database at the National Center for Biotechnology Information.

b Percentages are based upon a ClustalW alignment between the corresponding protein in *E. coli.*

### *S. meliloti* and *Ca.* Liberibacter Asiaticus *znu* Alleles Complement Δ*znu E. coli* Strains

A previous study involving solute-binding, protein–dependent transporters in *S. meliloti* 1021 reported a 37-fold induction upon zinc limitation of the gene SMc04245 (referenced above as ZnuA) [28], providing indirect evidence that the Znu system is functional in this bacterium. However, no direct evidence pertaining to the function of ZnuA or the other Znu proteins has been reported for *S. meliloti* nor have any studies been published to date indicating that either of the two *Ca.* Liberibacter asiaticus systems is operational.

**Figure 3:**
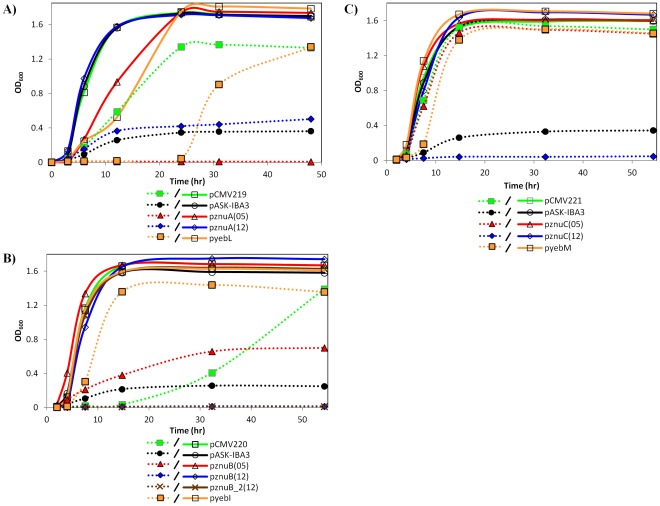
Growth of *E. coli* complemented strains. Strains of *E. coli* with **A)** *znuA,* **B)** *znuB, or* **C)** *znuC* insertionally inactivated were complemented with the corresponding *znu* genes from either *S. meliloti* (green), *E. coli* (orange), or *Ca.* Liberibacter asiaticus (red, blue, and brown) or the vector control (black). Strains were grown both in the absence (solid lines) and presence (dotted lines) of 0.4 mM EDTA. The absorbance at 600 nm (OD_600_) was measured at the time points indicated. Graphs are representative of data from three independent experiments.

To understand the role of the *Ca.* Liberibacter asiaticus and *S. meliltoi* Znu homologues, a complementation assay was devised to allow the individual genes that comprise the system to be assayed independently for their ability to re-establish the function of a partially-inactivated Znu system. *E. coli* was used as a heterologous host for these assays since a viable genetic system for *Ca. *Liberibacter asiaticus is not currently available *in vitro*. Here, three strains of *E. coli* containing an insertional inactivation of either *znuA*, *znuB*, or *znuC*, were transformed with a plasmid containing the respective *znu* allele from either *S. meliloti* or *Ca.* Liberibacter asiaticus **(** **Table 1** **)**. The alleles were placed under the control of a tetracycline-inducible promoter in order to eliminate any effects that the native promoters might have on overall transcript levels. Each putative gene comprising the Znu system was then tested independently for its ability to restore the high-affinity zinc uptake system in the appropriate *E. coli* knock-out strains by placing it in an inducing media that was depleted for zinc through the addition of 0.4 mM ethylenediaminetetraacetic acid (EDTA) and measuring the growth of the culture **(** **Fig. 3** **)**. The vector backbone (black lines/symbols) was transformed into the individual strains for use as a negative control as well as the respective native *E. coli* alleles (orange lines/symbols), which were used as positive controls.

All strains reached an OD_600_>1.5 when grown in TY **(** **Fig. 3** **, solid lines)** although the lag phase was consistently longer for the strains containing *znuA* from gene cluster #1 of *Ca.* Liberibacter asiaticus **(** **Fig. 3A** **, solid red line)** and *E. coli* **(** **Fig. 3A** **, solid orange line)**. Even though the final OD_600_ value was not as high when strains were grown in TY +0.4 mM EDTA, strains containing *znuA*, *znuB*, and *znuC* from *S. meliloti* **(** **Fig. 3A–C** **, dotted green lines)** appeared to complement the growth defect seen when only the vector control was present **(** **Fig. 3A–C** **, dotted black lines)**. These strains reached an OD_600_ that was similar to the *E. coli* controls **(** **Fig. 3A–C** **, dotted orange lines)**. Furthermore, the strain containing *znuC* from gene cluster #1 of *Ca.* Liberibacter *asiaticus* **(** **Fig. 3C** **, dotted red line)** was also able to grow to an equivalent density. Interestingly, the OD_600_ of the strain expressing *znuB* from gene cluster #1 of *Ca.* Liberibacter asiaticus **(** **Fig. 3B** **, dotted red line)** was less than cultures containing the *E. coli* or *S. meliloti* alleles but more than 2.5-fold greater than the vector control strain. This strain also showed dense growth on TY plates containing 0.4 mM EDTA compared to the vector control (data not shown), suggesting that it can complement the Δ*znuB* phenotype. Neither of the two *znuA* genes from *Ca.* Liberibacter asiaticus **(** **Fig. 3A** **, dotted red and blue lines)** appeared to complement the Δ*znuA* strain. In addition, neither the *znuB* **(** **Fig. 3B** **, dotted blue and brown lines)** nor the *znuC* genes from gene cluster #2 of *Ca.* Liberibacter asiaticus **(** **Fig. 3C** **, dotted blue line)** were able to complement the system and may in fact be detrimental to the growth since the OD_600_ of these strains were below those of the vector control.

### Growth Inhibition of *E. coli* and *S. meliloti* by EDTA

**Figure 4:**
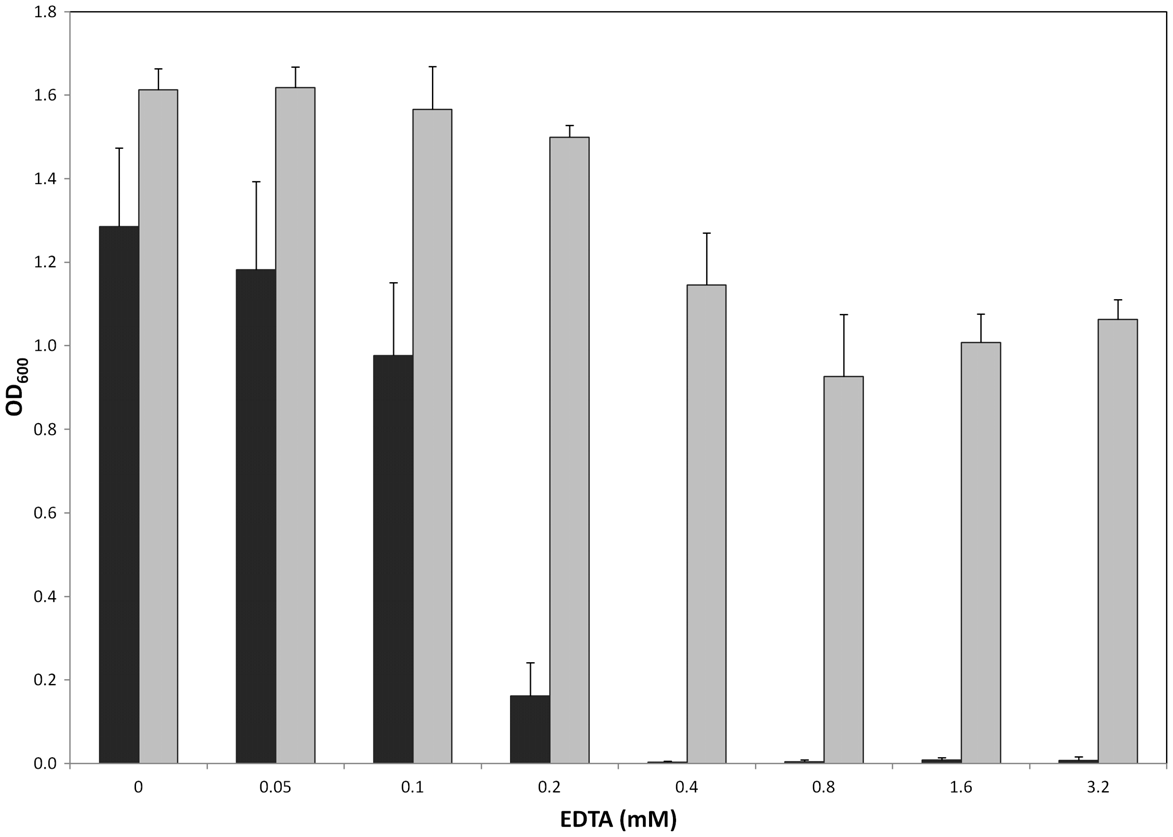
Inhibition of growth by EDTA. Increasing concentrations of EDTA was added to TY media inoculated with either *S. meliloti* (black bars) or *E. coli* (grey bars). The absorbance of the culture at 600 nm (OD600) was measured ∼20 hrs post inoculation. The average of three independent experiments is shown with error bars representing the standard deviation.

A concentration of EDTA as low as 0.2 mM was able to inhibit the growth of wild-type *S. meliloti* 1021 when added to the culture media although there were no negative effects on the overall growth of the wild-type *E. coli* strain at this concentration **(** **Fig. 4** **)**. This could indicate that either the *S. meliloti*’s Znu system is less effective than the *E. coli* system under low zinc conditions or, perhaps, *E. coli* possesses additional machinery for zinc uptake such as ZinT [29], [30], a protein involved in zinc uptake that is present in *E. coli* but not in *S. meliloti* [31]. However, when the *znu* genes of *S. meliloti* are expressed in a heterologous *E. coli* host those strains expressing the *S. meliloti* genes are able to grow in 0.4 mM EDTA **(** **Fig. 3** **),** which contradicts the hypothesis that the *S. meliloti* Znu system is less effective than the *E. coli* system.

### *S. meliloti* and *Ca.* Liberibacter Asiaticus *znu* Alleles Complement Δ*znu S. meliloti* Strains

**Figure 5:**
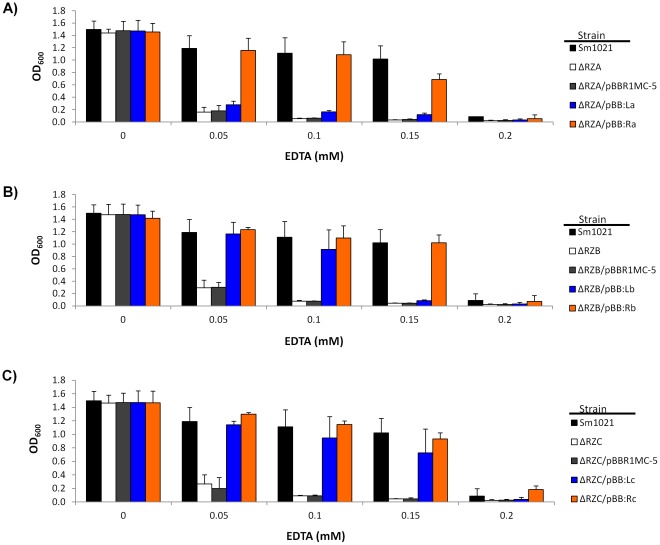
Growth of *S. meliloti* complemented strains. Deletion mutants of *S. meliloti* for **A)** *znuA* (ΔRZA), **B)** *znuB* (ΔRZB), or **C)** *znuC* (ΔRZC) were complemented with the corresponding *znu* genes from either the wild-type *S. meliloti* (orange bars), or *Ca.* Liberibacter asiaticus (blue bars). As controls, the wild-type *S. meliloti* strain (black bars), the uncomplemented deletion strains (white bars), and those containing the vector control (grey bars) were also grown. Strains were grown in different concentrations of EDTA ranging from 0 to 0.2 mM (x-axis). The absorbance at 600 nm (OD_600_) was measured at the time points indicated. Data shown is an average of three independent replicates with error bars representing the standard deviation.

Since the inhibitory concentration of EDTA varied significantly between *E. coli* and *S. meliloti,* complementation experiments were performed **(** **Fig. 5** **)** in knock-out *S. meliloti* strains (Δ*znuA,* Δ*znuB,* and Δ*znuC*) using the corresponding genes from the *Ca.* Liberibacter asiaticus gene cluster 1 or the native *S. meliloti* genes (the two operons that contained genes which were functional in *E. coli*). The results of Δ*znuC S. meliloti* strains paralleled those of the Δ*znuC E. coli* strains, with the *znuC* genes from both *S. meliloti* and *Ca.* Liberibacter asiaticus demonstrating the ability to complement **(** **Fig. 5C** **)**. Unique to the *S. meliloti* host strains was the fact that the *Ca.* Liberibacter asiaticus *znuB* gene appeared to restore the growth to relatively the same level as the *S. meliloti znuB* gene at the lower concentrations of EDTA **(** **Fig. 5B** **)** and the fact that the *Ca.* Liberibacter asiaticus *znuA* gene now appeared to partially compliment the Δ*znuA S. meliloti* strain at 0.1 and 0.15 mM EDTA **(** **Fig. 5A** **)**. All of the *znu* genes from *S. meliloti* were able to fully compliment, whereas the vector alone did not, as was predicted **(** **Fig. 5** **, orange bars vs. grey bars)**.

### Growth of *S. meliloti* in Zinc Repleated Media

**Figure 6:**
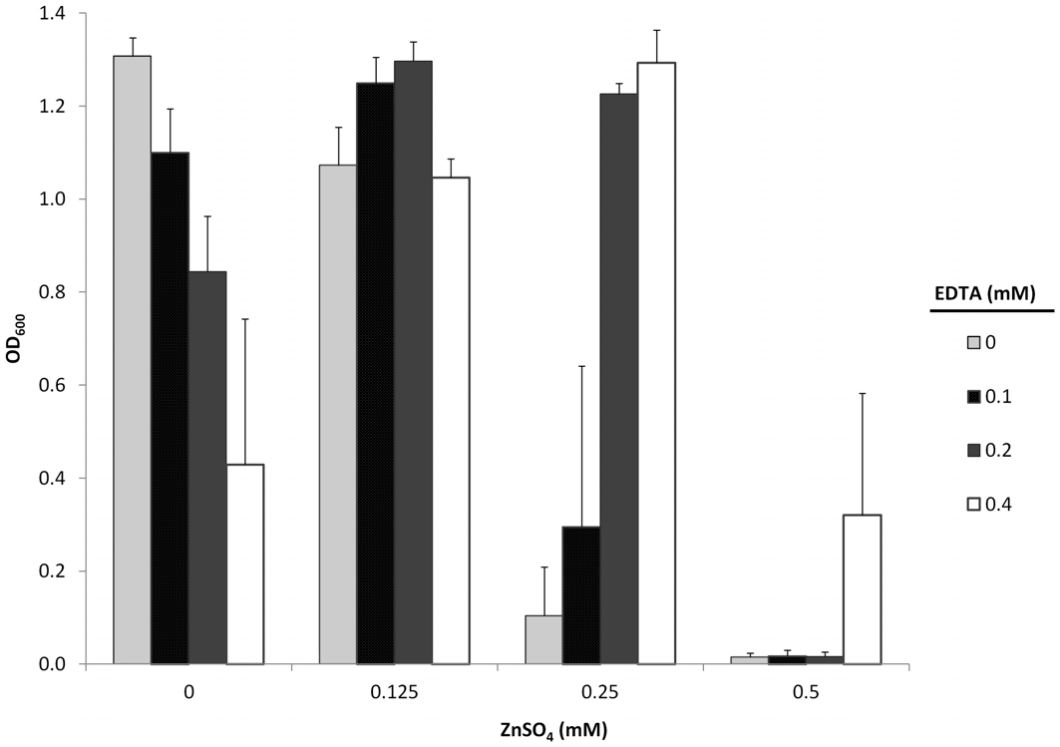
Growth of *S. meliloti* in zinc repleted media. Increasing concentrations of ZnSO4, ranging from 0 to 0.5 mM (x-axis), was added to media containing different amounts of EDTA (light grey bars: 0 mM, black speckled bars: 0.1 mM, dark grey bars: 0.2 mM, and white bars: 0.4 mM). The growth of *S. meliloti* in each media was determined by measuring the absorbance of the culture at 600 nm (OD600) ∼20 hrs post inoculation. Data shown is an average of three independent trials with three replicates grown per trial. Error bars shown represent the standard deviations.

Because EDTA is known to chelate other positively charged ions in addition to zinc, the growth of *S. meliloti* was observed in zinc repleated media to determine if the growth inhibition was the result of zinc chelation in the media **(** **Fig. 6** **)**. When wild-type *S. meliloti* 1021 was grown in TY media with various concentrations of EDTA (0, 0.1, 0.2, or 0.4 mM) that had been supplemented with different amounts of zinc (0, 0.125, 0.25, or 0.5 mM), a pattern emerged in which growth was inhibited under both high concentrations of EDTA without the addition of zinc **(** **Fig. 6** **-initial white bar)** and under high concentrations of zinc without the addition of EDTA **(** **Fig. 6** **-final black and grey bars)**. Given that growth could be restored in media containing 0.4 mM EDTA by the addition of zinc demonstrates the likelihood that the chelation of zinc by EDTA resulted in the diminished growth of *S. meliloti.* The growth of *S. meliloti* upon the addition of EDTA to media containing zinc concentrations that were previously inhibitive further supports this conclusion.

### Modes of Regulation in *S. melitoti* and *Ca*. Liberibacter Asiaticus

Once the function of several *znu* genes was established, putative modes of gene regulation were subsequently examined. A protein homologous to the Zur protein of *E. coli* was found immediately upstream of *znuB* in *S. melitoti,* though no homologues could be identified in the completed genome sequence of *Ca.* Liberibacter asiaticus using BLAST. The palindromic sequence to which Zur binds (Zur box) [31], which is commonly found within the promoter region of *znuA,* was also present only in *S. meliltoi* and not in the promoter region of either *znuA* genes in *Ca.* Liberibacter asiaticus (data not shown). In organisms such as *P. multocida,* transcriptional regulation of *znuA* has been shown to be under the control of the iron-uptake regulator, or Fur, instead of Zur [7]. However, a BLAST analysis of the *Ca.* Liberibacter asiaticus genome suggests that it does not contain a homologue of *fur* either. These data imply that the mode of regulation for *znuABC* could differ between *Ca.* Liberibacter asiaticus and *S. melitoti*.

## Discussion

### Complementation of the *znuA*, *znuB*, and *znuC* Alleles

Complementation experiments are an established method for assessing gene function in a biological system. Using this approach, we were able to define the functional role of several putative *znu* alleles from both *S. meliloti* and *Ca*. Liberibacter asiaticus. Despite only a modest degree of identity amongst the orthologous proteins and the difficulties involved in the execution of such studies because of the inability to reliably cultivate *Ca*. Liberibacter asiaticus, successful complementation assays were performed in two different host strains, both *E. coli* and *S. meliloti*. As a result of these studies, the role of the *znuA*, *znuB*, and *znuC* alleles in zinc uptake has now been demonstrated for the *S. meliloti* and *Ca*. Liberibacter asiaticus genes tested here.

**Figure 7:**
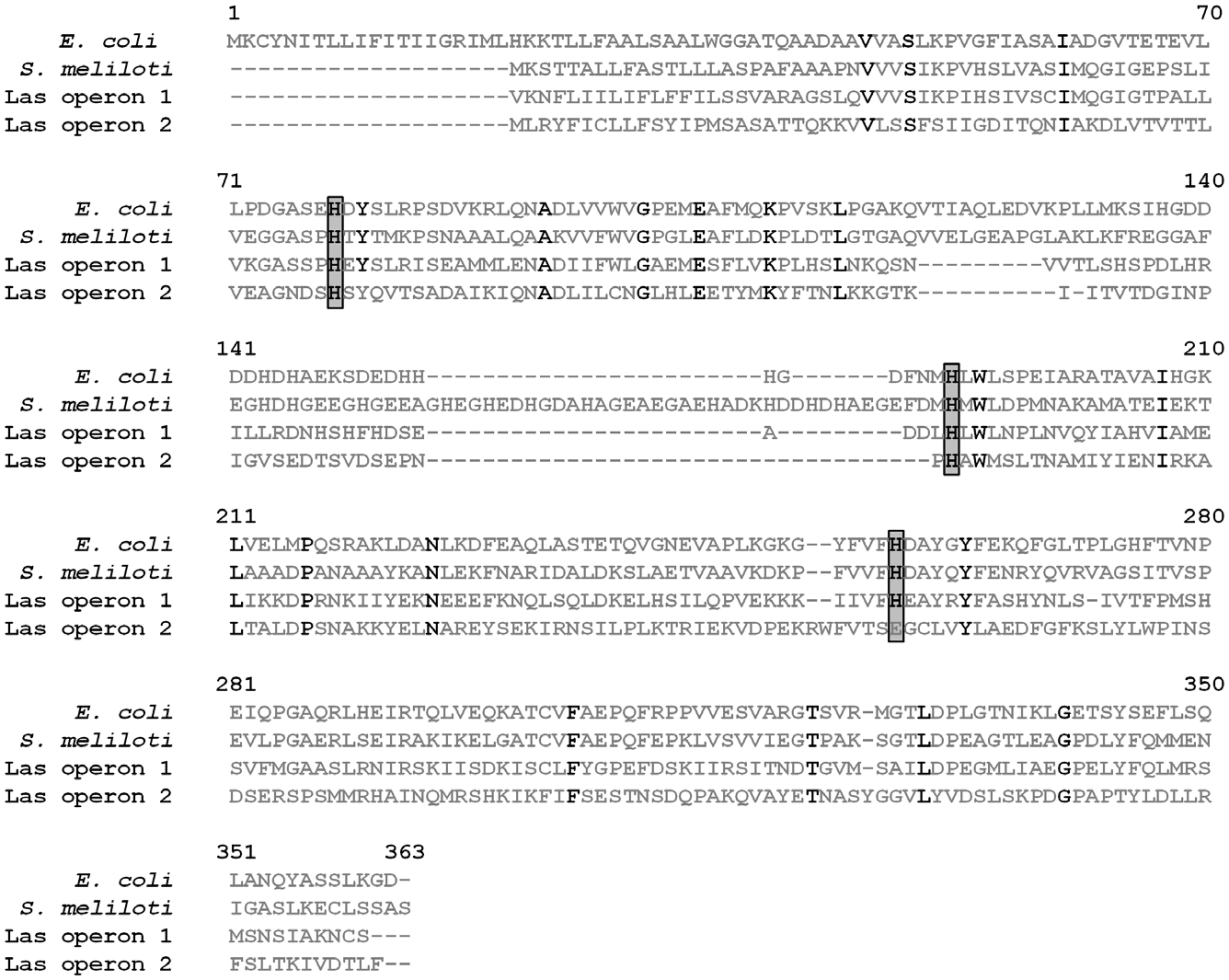
Sequence alignment of ZnuA orthologues. Orthologues to ZnuA from *S. meliloti* and *Ca.* Liberibacter asiaticus were aligned to ZnuA from *E. coli* using ClustalW. Residues that are 100% conserved amongst all orthologues are shown in bold. The three conserved His residues involved in binding zinc are shaded gray.

The biophysical mechanisms involved in the binding and transportation of the zinc by the Znu system has been characterized previously. Using the crystal structure, Banerjee *et al.* suggested that three conserved His residues were crucial for the binding of zinc in ZnuA [6]. A sequence alignment of the amino acid composition of ZnuA from *E. coli, S. meliloti*, and *Ca.* Liberibacter asiaticus revealed that ZnuA from the second gene cluster of *Ca.* Liberibacter asiaticus contained only two of the three conserved His residues **(** **Fig. 7** **)**, which may be a reason for its inability to complement **(** **Fig. 3** **)**. This apparent lack of complementation was not unexpected considering that none of the other Znu proteins from the second gene cluster of *Ca.* Liberibacter asiaticus appeared functional in the complementation assay.

Conversely, it was surprising that the ZnuA from the first *Ca.* Liberibacter asiaticus gene cluster only partially complimented the Δ*znu S. meliloti* strain **(** **Fig. 5A** **)** and did not complement the Δ*znu E. coli* strain **(** **Fig. 3A** **, dotted red line)** even though the other two genes present in that gene cluster were shown to be functional and all three His residues forming the zinc binding site were present **(** **Fig. 7** **)**. The lack of full complementation could mean the existence of a protein that works in conjunction with ZnuA to deliver zinc to membrane permease (ZnuB) or, perhaps, it is the result of the inability of ZnuA from *Ca.* Liberibacter asiaticus to be expressed properly/interact fully with the ZnuBC complex within *S. meliloti* or *E. coli*. This hypothesis is plausible considering ZnuA from *Ca.* Liberibacter asiaticus shares only 29% identity **(** **Table 2** **)** with *E. coli* (where a complete lack of complementation was observed) but 34% identity with *S. meliloti* (where partial complementation was obtained). Similarly, when the plasmid expressing *znuB* from gene cluster #1 of *Ca.* Liberibacter asiaticus was placed in the Δ*znuB E. coli* strain **(** **Fig. 3B** **, dotted red line)**, growth was only partially restored compared to the strain containing the *S. meliloti znuB* gene **(** **Fig. 3B** **, dotted green line)**, although when the same genes were placed in the Δ*znuB S. meliloti* strain **(** **Fig. 5B** **)**, both the *Ca.* Liberibacter asiaticus gene and the *S. meliloti* gene appeared to restore growth to levels similar to wild type at lower concentrations of EDTA. The higher percent identity that exists between the proteins from *Ca.* Liberibacter asiaticus and *S. meliloti* compared to *Ca.* Liberibacter asiaticus and *E. coli* implicates protein-protein interactions as a just cause for these discrepancies, however, when each of the two gene clusters from *Ca.* Liberibacter asiaticus was transformed into the Δ*znuA E. coli* strain in its entirety, the strains still did not outgrow the vector control in the presence of 0.4 mM EDTA (data not shown). Because the genes were under their native promoters in this construction, it is possible that not all of the genes were transcribed. It is also possible that the ZnuA gene from the second gene cluster interacts with the ZnuBC complex from the first *Ca.* Liberibacter asiaticus gene cluster but not with the ZnuBC complex from *E. coli*, which would also yield a negative result in this assay.

It is interesting to note that the structure and composition of both *Ca.* Liberibacter asiaticus gene clusters are highly conserved in a related species known as *Candidatus* Liberibacter solanacearum **(** **Table 3** **)** [32], the causal agent of zebra chip disease in potatoes. If these genes were completely non-functional, the high degree of conservation seen between the two would not be likely considering the greatly reduced genome sizes of these organisms and their different host ranges. Therefore, it is likely that these genes are functional, although how they function remains undefined. It is worth mentioning that the ZnuA protein from *Ca*. Liberibacter asiaticus gene cluster #2 is 33% identical to the known Mn^2+^ transporter, PsaA, from *Streptococcus pneumoniae* (Genebank accession # AAC24470.1) [33], [34]. Moreover, because the *Ca*. L. asiaticus ZnuA protein from gene cluster #2 contains a Glu **(** **Fig. 7** **)**, a residue predicted to facilitate Mn^2+^ binding [35], instead of a His at a position implicated in the binding of a zinc ligand [6], [36], it is possible that this protein is involved in the uptake of metals other than zinc.

**Table 3:** Sequence comparison of the Znu proteins from *Ca.* Liberibacter asiaticus vs. *Ca.* Liberibacter solanacearum.

Name	Genbank Sequence Record Number		% Identity^a^
	*Ca.* Liberibacter asiaticus	*Ca.* Liberibacter solanacearum	
ZnuA #1	ACT57190.1	ADR51883.1	63
ZnuA #2	ACT57010.1	ADR52867.1	65
ZnuB #1	ACT57192.1	ADR51885.1	82
ZnuB #2	ACT57013.1	ADR52870.1	77
ZnuB_2 #2	ACT57012.1	ADR52869.1	72
ZnuC #1	ACT57191.1	ADR51884.1	84
ZnuC #2	ACT57011.1	ADR52868.1	77

a Percentages are based upon a ClustalW alignment between the corresponding proteins.

### Growth Inhibition from Znu Expression

The expression of several *znu* genes from *Ca.* Liberibacter asiaticus, including *znuA* from the first gene cluster and both *znuB* and *znuC* genes from the second gene cluster, appears to be detrimental to the growth of the *E. coli* cultures in which the genes are expressed since the density of these strains when grown in TY + EDTA were below those of strains containing the vector alone **(** **Fig. 3** **)**. There are several explanations for this phenomenon. For example, over-expression of these proteins may be eliciting the death of the cell although this is not likely because the growth defect is only seen when the cells are grown in TY + EDTA and not in TY alone. In these assays, the level of expression is determined by the amount of anhydrotetracycline in the media not by the depletion of zinc through the addition of EDTA because the genes were placed under an inducible promoter. This makes the expression levels identical under the two conditions, yet the growth phenotype is only seen under the one condition. Another possibility is that these genes, whose functions do not appear to be related to the Znu system, may be producing proteins that are toxic to the cells once EDTA has chelated the metals in the system. These proteins could work by inhibiting one of the other zinc uptake systems in *E. coli,* such as ZupT [37], which may be responsible for the slight increase in culture density seen with the vector control. They could also be importing substances detrimental to growth under these conditions. Further characterization of these strains is necessary in order to determine the reason for their substantial lack of growth.

### Regulation of Zinc Uptake

The possibility of *Ca.* Liberibacter asiaticus and *S. meliloti* having distinct modes of regulation for the Znu system is intriguing given the close phylogenetic relationship [9] and the intracellular-plant niche common to both of these bacteria. Although the Znu system in *S. meliloti* appears to be regulated by Zur, alternative metal-sensing transcriptional regulators found within the cell may be able to regulate the expression of the zinc transporter genes since *Ca.* Liberibacter asiaticus lacks both *zur* and *fur,* the two currently known regulators of the Znu system [5], [7], [38]. It is also possible, given the obligate-intracellular nature of *Ca.* Liberibacter asiaticus, that the zinc level within host’s cells remain consistently low so the need for regulation has diminished and *znu* expression has become constitutive over time. Proteins involved in cation efflux, such as the metallothionein-like protein (GI: 254780343), could then be used to protect the bacterium against metal toxicity caused by small aberrances in intercellular zinc levels. Conversely, since an evolutionary trend towards constitutive expression of the Znu system would not be a viable mechanism in *S. meliloti* because of its existence as both a plant intracellular bacterium and an extracellular soil-borne bacterium, regulation via Zur was retained.

### Use of Znu System as a Target for HLB Control

Because zinc deficiency is already one of the most widespread deficiencies in citrus [39], possession of the Znu system by *Ca.* Liberibacter *asiaticus* and the subsequent uptake of zinc from its host may be resulting in a localized zinc deficiency in the citrus plant, which can ultimately lead to the death of the host’s plant tissues. This hypothesis is corroborated by the fact that the symptoms of plants affected by HLB often mimic those of a plant deficient in zinc and that the latter stages of HLB are characterized by twig dieback. Since Znu mutants have been shown to be attenuated for virulence in a variety of pathogens such as *Haemophilus ducreyi* [40], *Brucella abortus* [41], uropathogenic *E. coli* [42], and *Campylobacter jejuni* [43], it could be expected that mutations in this system would also affect the virulence of *Ca.* Liberibacter asiaticus. Thus, disruption of this system may present a viable target to not only lessen HLB symptom severity by making more free zinc available to the plant but also by providing a means to decrease the overall virulence of the bacterium itself. In addition, its lack of redundancy greatly increases the viability of such an option.

In conclusion, our findings clearly indicate a role in zinc uptake for the ZnuA, ZnuB, and ZnuC proteins from *S. meliloti* as well as one homologous gene cluster in *Ca*. Liberibacter asiaticus, providing the first conclusive evidence of this system in these plant-associated intracellular bacteria. These data also indicates that two distinct modes of regulation may exist for this high-affinity zinc uptake system despite a common intracellular-plant niche and their close phylogenetic relationship. The genetic and functional characterization of the Znu system not only provides insight into a system important for the growth and survival of these organisms but also provides a potential mechanism by which they might be manipulated.

### Notes

Mention of trade names or commercial products in this publication is solely for the purpose of providing specific information and does not imply recommendation or endorsement by the U.S. Department of Agriculture.

## Supporting Information

Table S1 **Primers used in this study.**
(DOC)
