# Supplementary material for: Two Plant Bacteria, S. meliloti and Ca. Liberibacter asiaticus, Share Functional znuABC Homologues That Encode for a High Affinity Zinc Uptake System
Source: PLoS One. 2012 May 24;7(5):e37340. doi: 10.1371/journal.pone.0037340 (PMC3360030; doi:10.1371/journal.pone.0037340)
Supplement: Table S1 — Primers used in this study. (DOC) [file pone.0037340.s001.doc]

**Table S1: Primers used in this study.**

| Target | Primer Name | Sequence* |
| --- | --- | --- |
| ***E. coli*** | | |
| *znuA* | yebL-F1 | atggtaggtctcaaATGAAATGTTATAATATCACACTTCTCATAT |
|  | yebL-R2 | atggtaggtctcagcgctATCTCCTTTCAGGGAGCTCGCA |
| *znuB* | znuB(E. coli)-F1 | atggtaggtctcaaATGATTGAATTATTATTTCCCGGTTGGTT |
|  | znuB(E. coli)-R1 | atggtaggtctcagcgctGCTGGCCTGCTTTTTCATCATAC |
| *znuC* | znuC(E. coli)-F1 | atggtaggtctcaaATGACAAGTCTGGTTTCCCTGGAAAAT |
|  | znuC(E. coli)-R1 | atggtaggtctcagcgctTGAGCGATCATTTCCCCGACGC |
| ***Ca.* Liberibacter asiaticus** | | |
| *znuA* #1 | LasZnuA1.C-For | cttgggcccGTTTTTATGAAAGCTGGTGT |
|  | LasZnuA1.C-Rev | gcgctgcagCTCTTTCTTATTTTG |
|  | znuA(05)-F2 | atggtaggtctcaaatgGTGAAAAATTTTTTAATAATACTTATATTTC |
|  | znuA(05)-R2 | atggtaggtctcagcgctAGAACAGTTCTTAGCTATTGAATTAG |
| *znuA* #2 | znuA(12)-F2 | atggtaggtctcaaATGTTACGTTATTTTATATGTTTGTTGTTCT |
|  | znuA(12)-R2 | atggtaggtctcagcgctAAATAAAGTATCCACAATTTTCGTTAG |
| *znuB* #1 | LasZnuB1.C-For | atagggcccTCCTCAAACCATCAAG |
|  | LasZnuB1.C-Rev | ctgctgcagGCGCTTGGCTATCCTATTTAA |
|  | znuB(05)-F2 | atggtaggtctcaaATGTATAATGAATTCTTCATTCGTGCCC |
|  | znuB(05)-R2 | atggtaggtctcagcgctTTTTTTATAGAAAAAGCTTAATATAAATAAG |
| *znuB #*2 | znuB(12)-F2 | atggtaggtctcaaATGATAAATCAAATTTTAGAACCTTTTACATA |
|  | znuB(12)-R2 | atggtaggtctcagcgctTGAGGTTCTGTATTGTTTAGAGAAAA |
| *znuB_2* #2 | znuB_2(12)-F2 | atggtaggtctcaaATGAGTAGTTTCCTACATTATGTTCTGTT |
|  | znuB_2(12)-R2 | atggtaggtctcagcgctGGCAGTTCTTTTATTGATAATTATTGA |
| *znuC* #1 | LasZnuC1.C-For | ttcgggcccTCATTAAGCTGCCACTTTTTGT |
|  | LasZnuB1.C-Rev | cgtctgcagATAATAAAGCATCCAAGCGGTC |
|  | znuC(05)-F2 | atggtaggtctcaaATGTCTAACGCCCTATCTATTACGCC |
|  | znuC(05)-R2 | atggtaggtctcagcgctGTGATCATGTTTGTGATTATGAATAG |
| *znuC* #2 | znuC(12)-F2 | atggtaggtctcaaATGAAAACAGCTGTCGCTTCCACGAA |
|  | znuC(12)-R2 | atggtaggtctcagcgctGCTTTTTTTTATGCACATGGTGGAA |
| ***S. meliloti*** | | |
| *znuA* | RmZnuA.C-For | tttgggcccACTGGCTATCGCACACGTTAT |
|  | RmZnuA.C-Rev | tgggaattcAAATGCCGGGACTAAACACT |
|  | znuA M-For | CAATGTGGTCGTCTCGATCAAG |
|  | znuA M-Rev | TACTGATAGGCATCGTGGAAGA |
|  | znuA(S. Mel)-F1 | atggtaggtctcaaATGAAATCGACGACTGCCCTCCTGT |
|  | znuA(S. Mel)-F2 | GCCTCCCCTCATACCTACACCA |
|  | znuA(S. Mel)-R2 | atggtaggtctcagcgctGCTCGCCGAAGAAAGGCATTCC |
|  | znuA(S. Mel)-R3 | GCACCTGGTAGCGGTTTTCG |
| *znuB* | RmZnuB.C-For | tttgggcccGACAACATTCATGACCACGATCC |
|  | RmZnuB.C-Rev | aagaattcATGCGGGCAGCTGCAGGCCACGA |
|  | znuB.M-For | GAAAGTCGACGACGGCAAAAAC |
|  | znuB.M-Rev | TCTTCGTGATGATGGCCGTC |
|  | znuB(S. Mel)-F1 | atggtaggtctcaaATGCTTGACGATTTCTTCATTCGCGC |
|  | znuB(S. Mel)-F2 | CCTCGTCATCGTCGCCTTCAT |
|  | znuB(S. Mel)-R2 | atggtaggtctcagcgctGTGATGTCCTCCATGCGAAGTAT |
|  | znuB(S. Mel)-R3 | TCCTCCATGCGAAGTATGCGG |
|  | znuB(S. Mel)-R4 | ATGAAGGCGACGATGACGAGG |
| *znuC* | RmZnuC.C-For | tttgggcccGGCTTGATCGAGACGACCACATT |
|  | RmZnuC.C-Rev | gttgaattccATGAGCAGCGAGAGCG |
|  | znuC.M-For | CTGCTCATGGACCTCAACCTCA |
|  | znuC.M-Rev | GCAGCAGACTCAGCACGAAAAG |
|  | znuC(S. Mel)-F1 | atggtaggtctcaaATGGTCAGCCTCGCCAATGCCGG |
|  | znuC(S. Mel)-F2 | CGCTTAAAGGTCGGGAAATCG |
|  | znuC(S. Mel)-F3 | GACGGCAGCATCACTGAAAGC |
|  | znuC(S. Mel)-R1 | atggtaggtctcagcgctAGCATCGCGCTTCTCCGTCCC |
|  | znuC(S. Mel)-R3 | GCTTTCAGTGATGCTGCCGTC |

* Noncomplementary sequences are in lowercase letters
